# A Sample-In-Answer-Out Microfluidic System for the Molecular Diagnostics of 24 HPV Genotypes Using Palm-Sized Cartridge

**DOI:** 10.3390/mi12030263

**Published:** 2021-03-04

**Authors:** Rui Wang, Jing Wu, Xiaodong He, Peng Zhou, Zuojun Shen

**Affiliations:** 1 Deparment of Clinical Laboratory, Anhui Provincial Hospital Affiliated to Anhui Medical University, Hefei 230001, China; WangR4171@163.com (R.W.); wujing950829@163.com (J.W.); 2 Deparment of Clinical Laboratory, The First Affiliated Hospital of USTC, Division of Life Sciences and Medicine, University of Science and Technology of China, Hefei 230001, China; h_xd5431@sina.com; 3 Beijing Bohui Innovation Biotechnology, Changping District, Beijing 102200, China; zhoup@advion.com

**Keywords:** sample-in-answer-out, microfluidic, human papillomavirus (HPV), reverse dot hybridization

## Abstract

This paper proposes an automated microfluidic system for molecular diagnostics that integrates the functions of a traditional polymerase chain reaction (PCR) laboratory into a palm-sized microfluidic cartridge (CARD) made of polystyrene. The CARD integrates 4 independent microfluidic sample lanes, which can independently complete a sample test, and each sample lane integrates the 3 functional areas of the sample preparation area, PCR amplification area, and product analysis area. By using chemical cell lysis, magnetic silica bead-based DNA extraction, combined with multi-PCR-reverse dot hybridization with microarray, 24 HPV genotypes can be typing tested in CARD. With a custom-made automated CARD operating platform, the entire process can be automatically carried out, achieving sample-in-answer-out. The custom-made operation platform is developed based on a liquid handling station-type, which can automatically load off-chip reagents without placing reagents in CARD in advance. The platform can control six CARDs to work simultaneously, detect 24 samples at a time. The results show that the limit of detection of the microfluidic system is 200 copies/test, and the positive detection rate of clinical samples by this system is 100%, which is an effective method for detection of HPV.

## 1. Introduction

Human papillomavirus (HPV) is the most common sexually transmitted infection in the world [1,2,3]. Numerous studies have confirmed that HPV infection causes a variety of cancers and precancerous lesions [4,5,6,7,8]. However, there are multiple genotypes of HPV, and more than 120 genotypes have been identified, according to their carcinogenic potential, they can be divided into high-risk HPV (hrHPV) and low-risk HPV (lrHPV) [9,10,11]. Typing tests for patients with HPV infection have always been a huge challenge for clinical diagnosis.

With the continuous development of molecular diagnostic technology, nucleic acid (NA) probe hybridization has become the mainstream method for the detection of HPV [12,13,14]. Hybrid Capture 2 HPV DNA Test (HC2), sold by Qiagen (Digene Corporation Gaithersburg, MD, USA), is the earliest HPV test method approved by the Food and Drug Administration of USA (FDA). It is based on the principles of the liquid phase in situ hybridization and signal amplification, and has high sensitivity for the detection of HPV [15]. HC2 is easy to operate, but the test results lack specificity, and typing test of hrHPV cannot be performed, and there is a cross-reaction between hrHPV and lrHPV, which limits its use in clinical diagnosis [16,17,18].

The microarray-based reverse hybridization technology, solidifies the DNA probes on the surface of the support in an orderly manner by microprinting, then the polymerase chain reaction (PCR) products are hybridized with the probes in situ, and the hybrid products are revealed by enzymatic reactions or by using fluorophores [13,19]. The application of reverse hybridization based on DNA microarray in HPV typing test has high sensitivity and specificity compared with the existing HPV detection technology, which can be used for simultaneous detection of multiple genotypes in multiple samples [20,21,22,23]. However, current microarray assay needs to be performed by separate instruments, which are cumbersome and risk of sample contamination. Moreover, some of these instruments, such as fluorescence scanners for signal visualization, are expensive and bulky instruments that can only be obtained in well-equipped laboratories [24].

The emerging field of microfluidics, the so-called lab-on-a-chip (LOC) or micro total analysis system, integrates a variety of equipment functions of traditional laboratories into a palm-sized chip and interconnects the functional areas through microchannels, using microvalves, micropumps, micromixers, reaction chambers and detectors to automate complex operations [25,26,27]. Integrating various steps of the microarray assay into coordinated and miniaturized LOC devices has become the goal of the microarray communities [24]. The combination of microfluidics and microarray technology achieves less sample and reagent usage and reduced incubation time, and the high surface area-to-volume ratio in the nano-solution volume microchannel dramatically improves the hybridization sensitivity [28,29,30].

Recently, it has become a trend to integrate all the steps of molecular diagnosis, including sample preparation, target nucleic acid purification and amplification, and result analysis, into a LOC system to achieve complete integration of sample-to-answer [31,32]. However, integrating all assay steps in a single device remains a challenge. A few commercial microfluidic platforms for nucleic acid analysis can realize the integration of several assay steps, not all steps. Such as Filmarray, which is a powerful system for multiple pathogen detection, that uses nested PCR and many individual microwells to detect multiple targets [33]. However, additional equipment is required for sample loading or sample preparation. Similarly, the Handylab’s cartridge and Cepheid’s GeneXpert, still requires some off-chip reagent operations or sample preparation [34]. In general, a truly practical LOC system needs to be fully automatic, easy to use, as well as sample-in-answer-out.

In this paper, we proposed a fully automated microfluidic system to analyse HPV clinical samples in disposable microfluidic cartridges (CARD) made of polystyrene (PS). The microfluidic system integrates multiple functions of traditional PCR laboratories. Use patented welding technology to integrate all pumps, valves, microchannels, and reaction chambers into CARD [35,36,37]. With the custom-made liquid handling station-style operating platform, cell lysis, DNA extraction with silicon-based magnetic beads, multi-PCR, and reverse dot hybridization with DNA microarray can be automated in CARD to perform qualitative detection of 24 HPV genotypes, achieving the detection goal of sample-in-answer-out. Besides, as the experiment proceeds, the off-chip reagents are added automatically without placing reagents in the chip in advance, which greatly reduces the difficulty and cost of CARD manufacturing. Using this microfluidic system, 24 samples can be automatically detected within 4.5 h, while testing one sample only costs about RMB 10.

## 2. Materials and Methods

### 2.1. Design of the Microfluidic System

**Figure 1:**
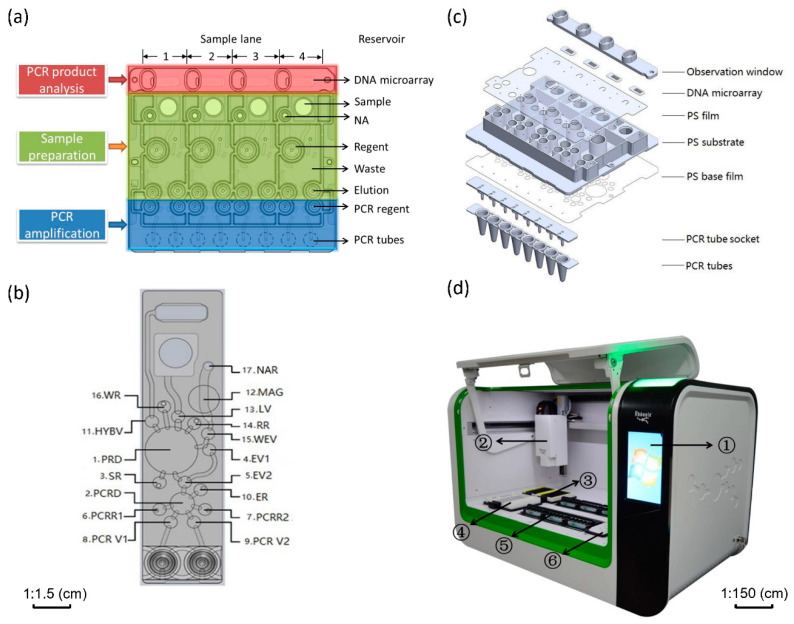
Illustration of the microfluidic system. (**a**) Schematic of CARD. A CARD contains 4 sample lanes, and each sample lane can be divided into 3 functional areas, a sample preparation area (green area), a PCR amplification area (blue area), and a PCR product analysis area (red area). (**b**) The structures contained in a sample lane. 1.PRD (Purification drive), 2.PCRD (PCR drive), 3.SR (Lysis reservoir), 4.EV1 (Elution valve 1), 5.EV2 (Elution valve 2), 6.PCRR1 (PCR reservoir 1), 7.PCRR2 (PCR reservoir 2), 8.PCRV1 (PCR valve 1), 9.PCRV2 (PCR valve 2), 10.ER (Elution reservoir), 11.HYBV (Hybridization valve), 12.MAG (Magnet), 13.LV (Lysis valve)), 14.RR (Reagent reservoir), 15.WEV (Washing and elution valve), 16.WR (Waste reservoir), 17.NAR (NA reservoir). (**c**) The 3D exploded view of CARD. CARD is mainly consists of 7 parts: observation window, DNA microarrays, PS film, PS substrate, PS base film, PCR tube socket, and PCR tubes. (**d**) The complete prototype of the custom-made operating platform, ① Touch screen for running control programs and obtaining test results, ② the 3D mobile module loaded with the reagent addition module and CCD camera, ③ micropipette tips placement slot, ④ the tank for placing the kit reagents, ⑤ manifolds for CARDs, ⑥ sample placement slot.

In order to achieve sample-in-answer-out detection of HPV genotypes in clinical samples, we designed the microfluidic cartridge (CARD) as shown in Figure 1a. A CARD contains four sample lanes, each sample lane can independently complete a sample test, and each sample lane has 3 functional areas, including sample preparation area, PCR amplification area, and product analysis area.

Sample preparation area: Using the chemical cell lysis with silicon-based magnetic beads for DNA extraction, and then elute the DNA bound to the silicon-based magnetic beads.

PCR amplification area: The eluted DNA is transferred to the CARD PCR tubes for the PCR amplification cycle of the HPV target gene.

Product analysis area: The PCR amplified products are transferred to the hybridization chamber after denaturation, and hybridized with the DNA microarray to identify multiple HPV genotypes.

All fluid operations are performed by the microstructure on the back of the sample lane as shown in Figure 1b. The CARD is made of low-cost polystyrene (PS), and the CARD size is 80 mm (length) × 80 mm (width) × 9.9 mm (depth). CARD consists of 7 parts as shown in Figure 1c, including an observation window, four DNA microarrays for reverse dot hybridization assay, a PS substrate with blocking channels, two PS films, a PCR tube socket, and PCR tubes. The real image of CARD can refer to Figure S1.

In the production of CARD, the PS substrate, as shown in Figure 1c, is first manufactured by injection molding, and then the PS films are welded to the PS substrate using a patented weak solvent-based bonding technology to form sample reservoirs and waste reservoirs, and form CARD pumps/valves and microchannels on the bottom surface of the substrate, as shown in Supplementary Figure S2. Since reagents are not loaded in CARD in advance, the external control system is used to load off-chip reagents as the experiment progresses. Therefore, we designed 5 reservoirs for loading reagents on the PS substrate, and there is a hole at the bottom of each reservoir. The reagents added into the reservoirs flow into the microfluidic structure on the bottom through the holes, thus achieving a perfect world-to-chip connection. Similarly, we also designed another hole on the substrate to allow the waste generated during the experiment to flow into the waste reservoir. Next, the PCR tube socket is laminated on the PS substrate, and then the PCR tubes are buckled into the PCR tube socket. Finally, DNA microarrays containing probes of 24 HPV genotypes are embedded on the substrate, and an observation window is laminated to form a hybridization chamber. The CARD works with the assistance of a custom-made operating platform.

The custom-made operating platform with a liquid handling station design, as shown in Figure 1d. The platform contains three fundamental components, a pneumatic drive system for fluid control in CARD, a temperature control system, and a three-dimensional (3D) mobile manipulator. The pneumatic drive system adopts two composite manifold plates with multi-layer pneumatic circuits, which integrates pressure sensors, gas storage tanks, pneumatic valves, and control circuits, making the control of complex air circuits integrated, and at the same time miniaturizing the drive system. Under the control of the drive system, up to 6 CARDs can work simultaneously. The temperature control system includes PCR temperature control module and hybridization temperature control module, which are also integrated on the manifold plate of the drive system. As for the 3D mobile manipulator, it integrates reagent addition modules and a CCD camera (MER-132-30GM, Daheng Imaging, Beijing, China), which can realize the automatic loading and unloading of micropipette tips, sample loading, reagent distribution, and acquiring the image of the test result. We have also designed the control software based on Java language for the cooperation of various components and the corrected grayscale value (CGV) analysis of the image result. The CGV calculation formula (PSG means the grayscale of probe site, SP1/2/3 means the grayscale of 3 SP sites):

(1) CGV=300∗PSG/(SP1+SP2+SP3)

### 2.2. On-CARD Fluid Control

The CARD pumps/valves are pneumatically connected to the bottom manifold. When the fluid needs to pass through the microvalve or micropump, the pressure controller applies negative pressure to the diaphragm and the diaphragm deflects downward to open the microchannel. On the contrary, positive pressure is applied to make the diaphragm close to the PS substrate to block the microchannel. As shown in Supplementary Figure S3a, by connecting multiple diaphragm pumps/valves in series, sequentially driving multiple diaphragms will produce peristaltic pumping, which can achieve continuous delivery of fluid in the CARD microchannel. It is also possible to continuously reverse the driving sequence of the microstructure between the two reaction reservoirs or between the micropump and the reaction reservoir to achieve the purpose of mixing fluids. Besides, CARD uses two pumps as drive nodes, each pump is connected to multiple microchannels and microvalves. As shown in Supplementary Figure S3b, by changing the driving sequence, the micropump can pump fluid in multiple directions, providing a high degree of flexibility for flow control.

### 2.3. Microarray

**Figure 2:**
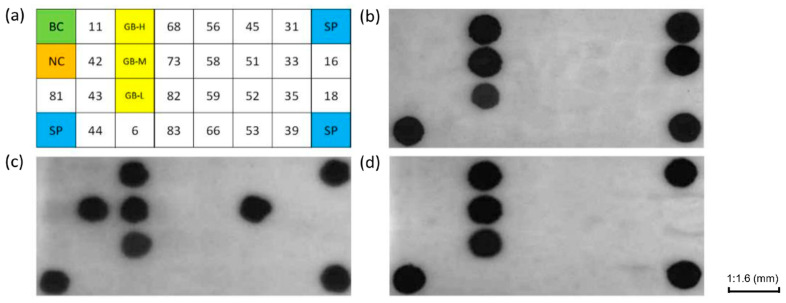
Microarray design and example of test results. (**a**) Illustration of probes distributed on a microarray, including 1 Blank control (BC, green area), 1 Negative control (NC, orange area), 3 internal reference control (GB, yellow area) with different concentrations, 3 Spot control (SP, blue area), HPV probes of 24 genotypes (18 hrHPV probes:16, 18, 31, 33, 35, 39, 45, 51, 52, 53, 56, 58, 59, 66, 68, 73, 82, 83; 6 lrHPV probes:6, 11, 42, 43, 44, 81. white area). (**b**) HPV16 positive results; (**c**) HPV42, 51 double-points positive results, (**d**) Negative result.

The DNA microarray for reverse dot hybridization assay to detect multiple genotypes of HPV is made of a negatively charged nylon membrane (PALL corporation, New York, NY, USA) with high-density carboxyl groups on the surface, which can bind the target to the surface of the membrane by covalent bond. The size of the DNA microarray is 9.6 mm (length) × 4.5 mm (width), and the thickness is 279.4–330.2 μm. The microarray contains 18 hrHPV probes and 6 lrHPV probes. As shown in Figure 2a, the dot matrix of HPV probes was set to 4 × 8 matrix, and the positions of probes were evenly distributed on the membrane. It can be used not only for single genotype detection, but also for detecting compound genotypes. Refer to Figure 2b–d for test results examples. The DNA microarray also has 3 Internal reference control (GB) probe sites, 3 Spot Controls (SP) probe sites, 1 Negative Control (NC) probe site, and Blank Control (BC) probe site. GB is a conservative sequence of human β-globin, and the 3 GB probe sites are 3 different concentrations of GB probes (GB-H 50 μM, GB-M 20 μM, GB-L 2 μM). In practice, when we use this microfluidic system for sample detection, sometimes the cell concentration in the sample is low, resulting in low human globin gene content, but as long as the test result is positive for GB-H, it means the extracted nucleic acid meeting the testing requirements, regardless of whether GB-M and GB-L are positive, there is no effect on the result determination. However, if the GB-H site is negative in the test result, it means that hybridization process may have failed, and the sample needs to be tested repeatedly. If GB-H is still negative in the result of repeated testing, it indicates that the sample collection does not meet the requirements. SP is a 19-base thymine sequence modified with 5′amino and 3′biotin to monitor the color development process. If the 3 SP points in the result are negative, it means that the color development has failed. NC is the same as that of the SP, except that there is no biotin modification at the 3′end of the sequence, while BC has no probe sequence and only contains probe diluent. If BC or NC in the result is positive, it means that contamination occurred during the experiment and the test failed. Only when the three SP sites and GB-H site on the microarray are positive, can the positive or negative results of other HPV genotype probe sites be reliably determined.

### 2.4. Primers and Probes

The different HPV genotypes sequences was obtained from the National Center for Biotechnology Information (NCBI). Each genotype selects 1 to 438 sequences. The ClustalX software is used for comparison, and the conserved genes in the HPV L1 region are selected for primer and probe design. Two upstream and downstream primers and three probes were designed for each genotype. By comparing the amplification efficiency of different primer combinations, the primer pair with the best amplification effect is screened out. One probe with the highest hybridization efficiency was obtained by comparing the brightness of hybridization spots and the contrast of hybridization spots. When the selection of the primer is done, the upstream and downstream primers of the genotype are degenerate to ensure that the 24 HPV genotypes are amplified and the number of sequences used at the same time is the least. Finally, 8 upstream primers and 7 downstream primers were determined. The primer and probe sequences can refer to Supplementary Tables S1 and S2.

### 2.5. Preparation of Samples and Regents

110 clinical samples of cervical swabs were provided by the First Affiliated Hospital of the University of Science and Technology of China to evaluate the clinical performance of the microfluidic system. The study was approved by the Hospital Ethics Committee of the first affiliated Hospital of the University of Science and Technology of China. Results of this study did not influence the clinical discussion between patient and physician. All samples were handled anonymously.

To use the microfluidic system for 24 HPV genotypes detection, a matching detection kit was designed. The primary reagents were composed of three types: NA extraction reagents, PCR amplification reagents, and reverse dot hybridization assay reagents. The main raw materials used in the experiment were HPV primers (including 8 forward primers and 7 reverse primers), internal reference primers designed by the conserved sequences of the β-globin region in human cells (Sangon Biotech, Shanghai, China). Taq DNA polymerase (PROMEGA, Madison, WI, USA, Go Taq DNA polymerase, 5 U/μL), dNTPs (PROMEGA, 100 mM), uracil DNA glycosylase (UDG) (PROMEGA, 1 U/μL), and Horseradish peroxidase (HRP) labeled streptavidin (Thermo, Waltham, MA, USA, 162 U/mg). The remaining reagents contained in the matching detection kit can refer to Supplementary Table S3.

### 2.6. Workflow of the Microfluidic System

**Figure 3:**
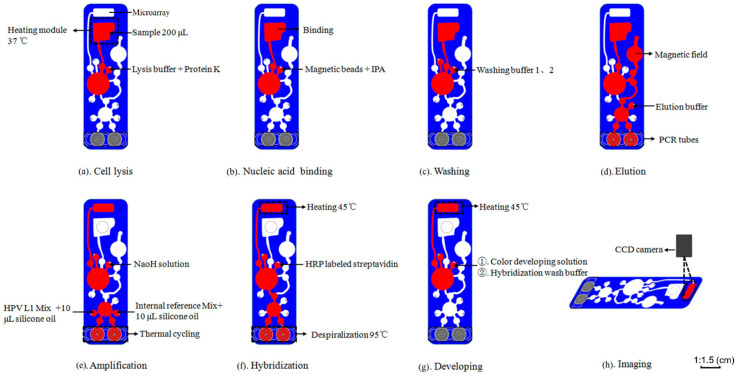
The CARD processing protocol. Illustration of flow control in CARD. Automatic sample detection process in CARD: (**a**) add lysis buffer and proteinase K to lyse cells, (**b**) magnetic beads binding DNA, (**c**) washing the DNA-bound magnetic beads with washing buffer, (**d**) add elution buffer to elute DNA, (**e**) add PCR amplification system for thermal cycling, meanwhile add membrane treating solution (NaOH) to activate the DNA microarray. (**f**) PCR amplification products are pyrolyzed into single strands and transfer to DNA microarray, then add HRP labeled streptavidin for incubation, (**g**) add color developing solution for color developing, then add Hybridization washing buffer to rinse the DNA microarray, (**h**) the CCD camera obtains image result.

Before the assay, put the reagents into the reagent preparation area, then introduce untreated samples directly into the CARD sample reservoir, finally start the detection program. Figure 3 details the HPV detection process. The principle of the detection process can refer to Figure S4. All the steps described are automatically performed under the control of the operating platform. The whole detection process is as follows:

Cell lysis: The lysis buffer and protease K were added into the reagent reservoir, pumped to the sample reservoir, and be pumped bi-directionally between the sample reservoir and PRD to mix, and the temperature was set at 37 °C to lyse the cells (Figure 3a).

DNA binding: Add silicon-based magnetic beads and isopropyl alcohol (IPA) to the reagent reservoir, and pump to the sample reservoir to bind DNA (Figure 3b).

Washing: Add washing buffer I to the reagent reservoir and pump it to the sample reservoir to remove residual inorganic salts and other small molecules, and then add washing buffer II to remove residual proteins, and then the waste is pumped into the waste reservoir (Figure 3c).

Elution: The DNA-bound magnetic beads are pumped to the NA reservoir, and then the elution buffer is added to the elution reservoir and pumped bi-directionally between the NA reservoir and the elution reservoir while attaching a magnet to the MAG. When the magnetic beads pass by, the magnetic beads are attracted to the MAG area by the magnet, and the DNA bound to the magnetic beads is eluted and pumped into PCR tubes to wait for PCR amplification (Figure 3d).

PCR amplification: Add HPV L1 amplification reagents and internal reference amplification reagents to PCR reservoirs 1 and 2, respectively, and then pump into PCR tubes. Next, add 10 μL of silicone oil to PCR reservoir 1 and 2, and pump into PCR tubes to prevent evaporation, and then start PCR amplification process (37 °C for 10 min, 95 °C for 2 min, then 10 cycles, 95 °C for 30 s, 46 °C for 30 s, and 72 °C for 30 s, next 30 cycles, 95 °C for 17 s, 49 °C for 30 s, and 72 °C for 30 s, finally, 72 °C for 3 min). At the same time, add membrane treating solution to the reagent reservoir and pump it to the DNA microarray to activate the DNA microarray (Figure 3e).

Reverse dot hybridization: Denature the amplified DNA to single-stranded DNA at 95 °C and pump it to DNA microarray and incubate at 45 °C. The single-stranded DNA hybridizes with the probe on the DNA microarray. Then add HRP labeled streptavidin to the reagent reservoir, pump to the DNA microarray (Figure 3f).

Color developing: Add the color development solution to the reagent reservoir, and pump to the DNA microarray. The biotin at the 3′end of the single-stranded DNA hybridized on the DNA microarray binds to HRP labeled streptavidin and reacts with the color developing solution. Next, add hybridization washing buffer to the reagent reservoir to rinse the DNA microarray (Figure 3g).

Imaging: Using the CCD camera to obtain the image and the software analyses the CGV of the image result to determine whether the result is positive (Figure 3h).

In addition, the above steps can be easily reprogrammed to meet other experimental protocols.

## 3. Results and Discussion

### 3.1. Characterization of Pumping Precision

**Figure 4:**
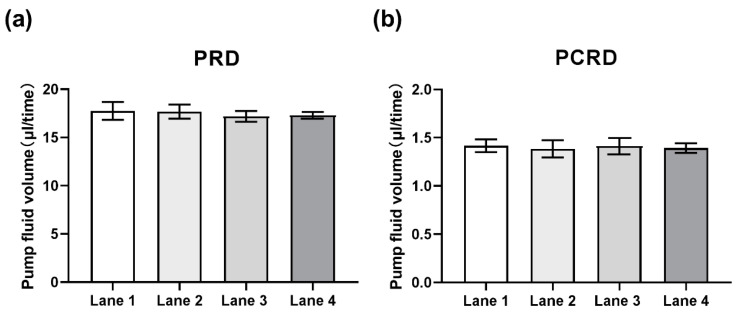
Characterization of pumping precision. (**a**,**b**) Under the preset working conditions (69 kPa, −55 kPa, 10 Hz), the PRD and PCRD of each sample lane pumped liquid volume at one time. Error bars represent standard deviation (*n* = 3).

The microfluidic system can automatically carry out cell lysis, DNA extraction, PCR amplification, and reverse dot hybridization assay, which requires micro-upgraded fluid operation, so there is a high requirement for the pumping precision of the whole microfluidic system. A sample lane uses two micropumps as driving nodes, so we select two typical flow paths of RR-SR and ER-PCRR2 as representatives, of which RR-SR is a PRD path, and ER- PCRR2 is a PCRD path. Under the preset working conditions (positive pressure 69 kPa, negative pressure −55 kPa, 10 Hz), test the pumping precision of PRD and PCRD. Put enough ddH_2_O into RR and ER, PRD pump 10 times, PCRD pump 50 times. Take ddH_2_O from SR and PCRR2 to weigh with an electronic balance and ANOVA on the results. The result is shown in Figure 4b, it is found that the CV of the PRD pumping fluid volume of the four sample lanes is 15.97%, and the CV of the PCRD pumping fluid volume is 21.55%. Based on the above results, we believe that it can fully meet the requirements of automated testing.

### 3.2. Optimization of Reverse Dot Hybridization Assay

**Figure 5:**
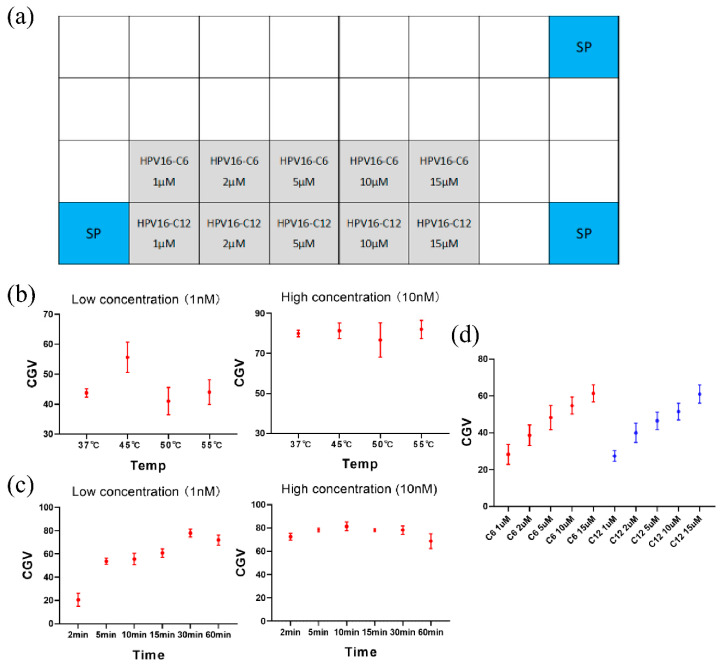
The CGV of the reverse dot hybridization under different conditions, the higher the CGV, the better the detection result. (**a**) Microarray is fabricated with two differently modified HPV16 genotype probes (HPV16-C6, HPV16-C12) at 5 different concentrations (1 μM, 2 μM, 5 μM, 10 μM, 15 μM). (**b**) The CGV results of samples with different concentrations under different hybridization temperature conditions. (**c**) The CGV results of samples with different concentrations under different hybridization time conditions. (**d**) The CGV results of two different modified probes with 5 different concentrations, when the sample concentration is 100 pM. Error bars represent standard deviation (*n* = 4).

The reverse dot hybridization assay is a key step in determining the HPV genotype in the sample. Therefore, we have optimized the operating conditions of reverse dot hybridization assay, including the optimal hybridization temperature and hybridization time. Meanwhile, we also tested the effects of different probe concentrations and different modified probes on the hybridization results. Considering that the HPV16 genotype is the most carcinogenic and most infectious genotype in the world, we chose HPV16 as a representative to optimize the hybridization process. We diluted the two modified HPV16 genotype probes (C6 and C12) to different concentrations (1 μM, 2 μM, 5 μM, 10 μM, 15 μM) to make the DNA microarray as shown in Figure 5a. Different concentrations of HPV16 genotype plasmid (10 nM, 1 nM) were absorbed 15 μL, added to the CARD PCR tubes, and then 10 μL silicone oil was added. Next, the PCR tubes were buckled into the PCR tube socket, and the CARD was placed on the manifold of the operating platform. The temperature was set to 37 °C, 45 °C, 50 °C, and 55 °C, and the time was set to 10 min. The CGV of the HPV16-C12 10 μM probe site on the DNA microarray was selected for data processing and analysis. According to the temperature optimization results, the temperature is set to the optimal temperature, and the time is set to 2 min, 5 min, 10 min, 15 min, 30 min, 1 h, other experimental conditions remain unchanged. Similarly, the CGV of HPV16-C12 10 μM probe site on DNA microarray was selected for data processing and analysis.

In the process of reverse dot hybridization assay under different conditions, it is found that the CGV of low concentration samples (1 nM) is higher than that of other temperatures when the temperature is 45 °C, but for high concentration samples (10 nM), the temperature change has little effect on the CGV. (Figure 5b) Therefore, it is considered that 45 °C is the optimal temperature for the reverse dot hybridization assay. When the temperature is 45 °C, the CGV of the low concentration samples is higher with the extension of time, and the CGV is the highest when the time is 30 min. However, when the time is 60 min, the CGV decreases (Figure 5c). This may be because the washing time is too long, which leads to the loss of microarray surface probes and the decrease of the number of binding to the single-stranded DNA. Similarly, for the high concentration samples, the effect of time on the CGV is not obvious, so it is considered that 45 °C, 30 min is the best condition for reverse dot hybridization assay. However, the 30 min reverse dot hybridization process greatly prolongs the time of the overall detection process. A truly practical detection system needs to achieve the best detection results in a short time. The CGV of 5 min, 10 min, and 15 min have met the detection needs, so in the final finished product system, we chose 45 °C, 10 min as the operating condition of reverse dot hybridization assay. In the case of obtaining the best reverse dot blot assay conditions, we tested the sample with a concentration of 100 pM, and analysed the result CGV of all probe sites on the microarray. The results show that the higher the probe concentration, the higher the CGV. However, there is little difference in CGV between C6 and C12 modified probes. (Figure 5d) In the subsequent experiments, C6 modified probes were used to make DNA microarrays.

### 3.3. Limit of Detection (LOD)

**Figure 6:**
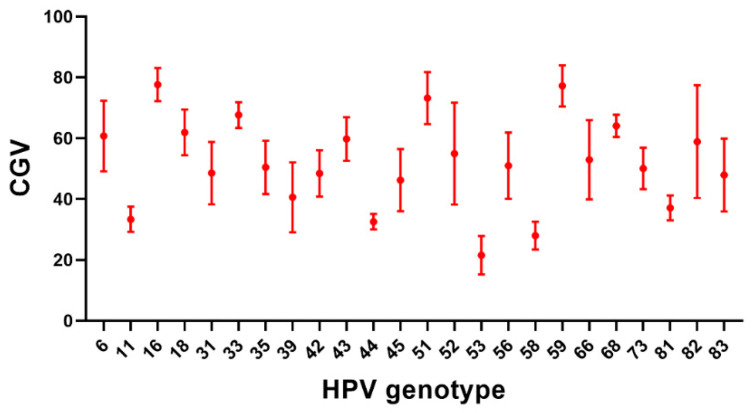
Test results of 24 simulated HPV genotype samples at the same concentration in the microfluidic system. The CGV results of 24 HPV genotypes while the sample concentration is 10^3^ copies/mL.

The LOD is an important indicator for testing the performance of a detection tool. To evaluate the LOD of the microfluidic system for detecting 24 HPV genotypes, we used the national standard references containing HPV plasmids of 24 genotypes (10^11^ copies/mL) were mixed with HEK293 cells as simulated samples, and diluted to 10^7^ copies/mL, 10^6^ copies/mL, 10^5^ copies/mL, 10^4^ copies/mL, 10^3^ copies/mL, 10^2^ copies/mL. Simulated samples were tested with detection kits, each concentration sample was tested 2 times, and quality control was performed with negative and positive quality control products. According to the optimal reverse dot hybridization assay conditions, the detection is completed on the microfluidic system, the image results are obtained by the CCD camera, and the CGV results are obtained by the analysis software. The results show that when the concentration is not less than 10^3^ copies/mL, the results for each genotype are positive. However, it is difficult to obtain stable positive results when the concentration is 10^2^ copies/mL, so we confirmed that the LOD of the microfluidic system is 10^3^ copies/mL. Figure 6 shows the CGV results of the 24 HPV genotypes when the sample concentration is 10^3^ copies/mL. Since multiple HPV genotype primers are degenerate when designing the primers, the same degenerate primer has different specificities for different HPV genotypes, resulting in differences in amplification efficiency. In the subsequent hybridization process, some genotypes have less amplified products resulting in large differences in CGV test results. It can also be seen from Figure 6 that the microfluidic system has different detection sensitivity for different HPV genotypes at the same sample concentration. However, the multi-genotype HPV test kits that are frequently used in clinical practice and approved by the National Medical Products Administration of China, such as Human papillomavirus nucleic acid genotyping kit (flow fluorescence hybridization method) (Tellgen Corporation, Shanghai, China) have a detection limit of about 100 copies/test; another example is the Human Papillomavirus Genotyping Kit For 23 Types (PCR-RDB) (Yaneng Biotechnology Co., Ltd., Shenzhen, China), with a detection limit of 10^3^ copies/mL. Compared with the above two kits, the microfluidic system should be able to meet the HPV multi-genotype clinical detection in most cases.

### 3.4. Evaluation of Methodology in Clinical Performance

In order to test the clinical performance of the microfluidic system, we used the system to test 110 cervical swab clinical samples from the First Affiliated Hospital of the University of Science and Technology of China. At the same time, 110 clinical samples of cervical swabs were tested using the Assay kit genotyping human papillomavirus (PCR-reverse dot blot) (LBP medicine science and technology co., Guangzhou, China) approved by National Medical Products Administration (NMPA), and the test results of our microfluidic system and the kit were compared. The microfluidic system detected 18 samples of hrHPV infection (including 5 cases of HPV52, 3 cases of HPV58, 2 cases of HPV59, 2 cases of HPV31, and 1 case each of HPV35, HPV33, HPV45) and 5 cases of lrHPV samples (including 2 cases of HPV42, 2 cases of HPV6 and 1 case of HPV44). At the same time, 4 cases of samples with compound genotypes were detected, including HPV66, 52, HPV42, 56, HPV52, 51, HPV44, 35. The remaining samples tested negative. The test result of the microfluidic system is consistent with the test result of the kit. The evaluation results show that the microfluidic system in this paper is suitable for HPV multi-genotype detection in clinical samples.

## 4. Discussion

HPV spreads widely around the world, especially for developing countries, the number of patients infected with HPV is increasing year by year [38,39]. Currently, commercial methods for HPV detection rely on PCR. However, the construction of a standardized PCR laboratory requires a lot of funds to purchase a variety of large-scale bench-top instruments, and requires professionals with relevant operating skills to operate, which is why the number of HPV infections in developing countries remains high. In order to avoid contamination, the standardized PCR laboratory needs to divide multiple areas (such as reagent preparation area, specimen preparation area, amplification area, product analysis area, etc.) to perform different functions. We have divided 3 modules in the disposable CARD, which can cover various functions of the PCR laboratory. Using the microfluidic technology, the waste liquid and amplification products produced in the detection process are all retained in the closed CARD reservoirs, which realizes spatial physical isolation from the external environment, effectively avoids the aerosol pollution of the samples and amplification products, greatly improves the accuracy of the test results and effectively ensures the safety of the experimental operators.

The reverse dot hybridization assay is the core step of HPV genotyping. The probes are designed with the conservative sequences of the L1 region of 24 HPV genotypes, and the probes are fixed on the membrane, and each probe has a point and position numbering. The 5′end of the PCR primer is pre-labeled with biotin, so that the amplified product is labeled with biotin, and then the product specifically amplified by PCR is hybridized with the microarray. The sample to be tested is bound to a probe with a homologous sequence, and the unbound DNA sample is removed by washing. Since the amplified product is labeled with biotin, the probe point that binds to the amplified product is labeled with biotin, and then the hybridization signal can be displayed by the color reaction. Its biggest feature is its simplicity, speed, and high resolution ability. In this article, we also optimized the hybridization process, and finally determined that hybridization with C6 modified probes at 45 °C for 10 min is the optimal operating condition.

The microfluidic system we developed that uses multiplex-PCR-reverse dot hybridization to detect 24 HPV genotypes in HPV samples within the developed disposable microfluidic CARD. The automated operation from HPV sample to answer in CARD is realized on the custom-made liquid handling station-style operation platform, and the off-chip reagent loading can be automated. The LOD of the system for the detection of 24 HPV genotypes was 10^3^ copies/mL. Moreover, it can be used for the detection of clinical samples and compared with the detection results of other products of the same type, with an accuracy rate of 100%. In addition, automated off-chip reagent loading and flexible flow control make the system have the possibility of applying existing commercial kits for other testing items to the microfluidic system for testing. Therefore, other detection projects are currently being developed on the system, and it can be used as a multi-functional platform for molecular diagnosis in the future.

## 5. Conclusions

The microfluidic system introduced in this article has considerable sensitivity and specificity for HPV typing detection, which can assist clinicians to make diagnoses and formulate corresponding treatment plans.

## Data Availability

The data that supports the findings of this study are available within the article and its Supplementary Material.

## Supplementary Materials

The following are available online at https://www.mdpi.com/2072-666X/12/3/263/s1, Figure S1: The real image of CARD, Figure S2: Principles of CARD microstructure production, Figure S3: Schematic diagram of on-CARD fluid control, Figure S4: Schematic diagram of the principle of detecting HPV, Table S1: Primer sequences of 24 HPV genotypes and internal reference (GB), Table S2: Probe sequences of 24 HPV genotypes, Table S3: The remaining reagents contained in the matching detection kit.
