# Supplementary material for: A Sample-In-Answer-Out Microfluidic System for the Molecular Diagnostics of 24 HPV Genotypes Using Palm-Sized Cartridge
Source: Micromachines (Basel). 2021 Mar 4;12(3):263. doi: 10.3390/mi12030263 (PMC8000143; doi:10.3390/mi12030263)
Supplement: Supplementary file 1 [file micromachines-12-00263-s001.pdf]

micromachines

MDPI

Supplementary Materials: A Sample-in-Answer-out Microfluidic System for the Molecular Diagnostics of 24 HPV Genotypes Using Palm-Sized Cartridge

Rui Wang, Jing Wu, Xiaodong He, Peng Zhou and Zuojun Shen

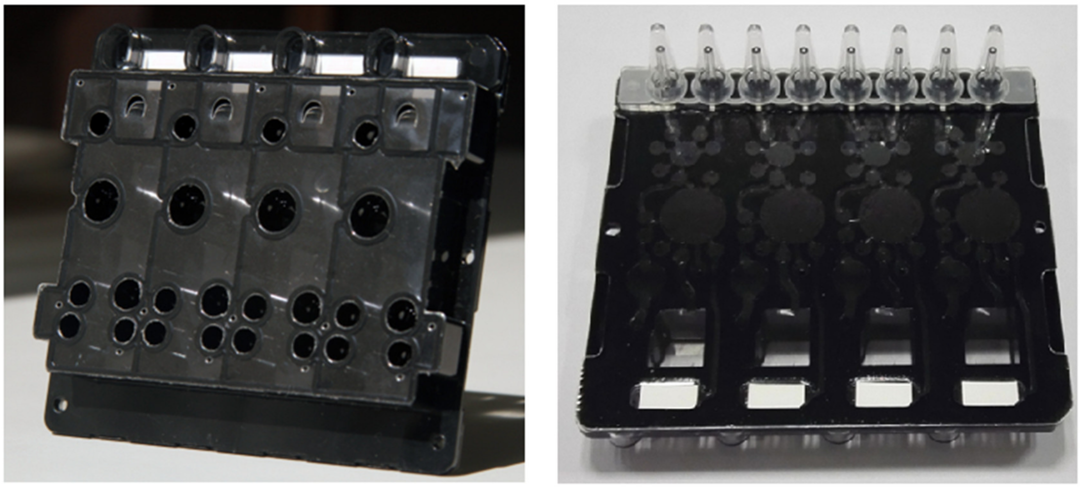

Figure S1. The real image of CARD.

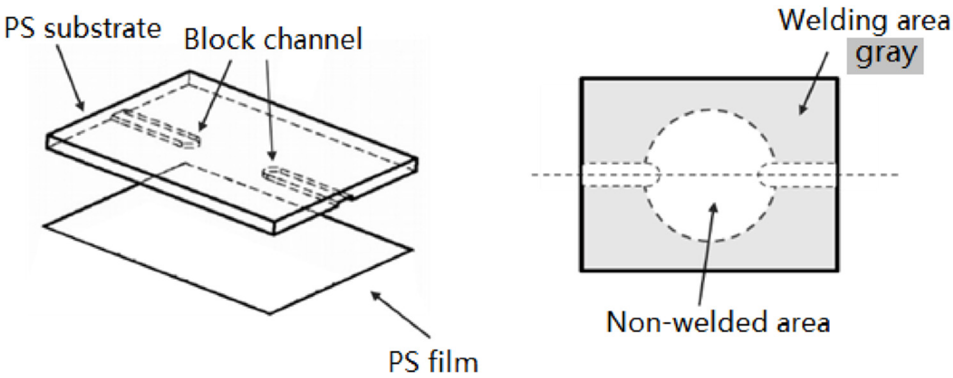

Figure S2. Principles of CARD microstructure production.

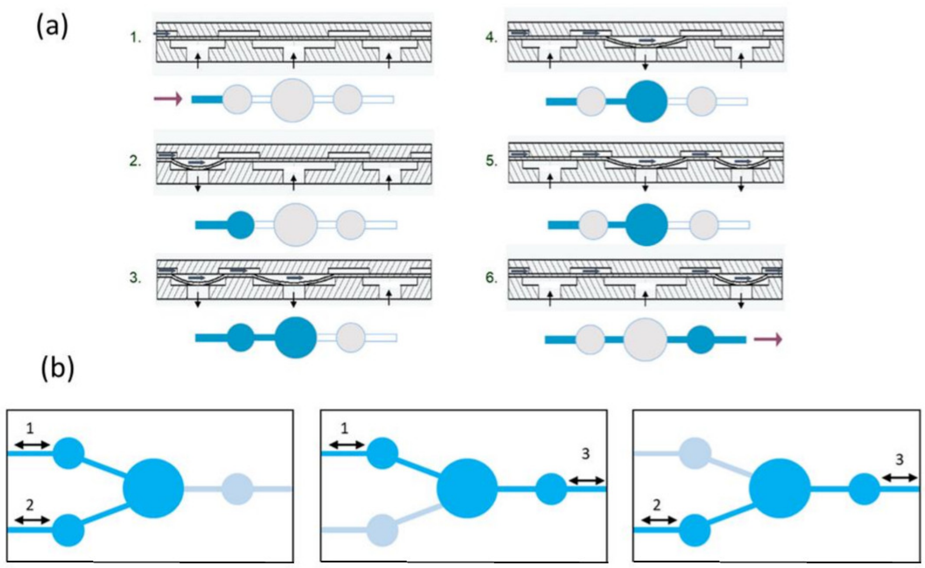

Figure S3. Schematic diagram of on-CARD fluid control.

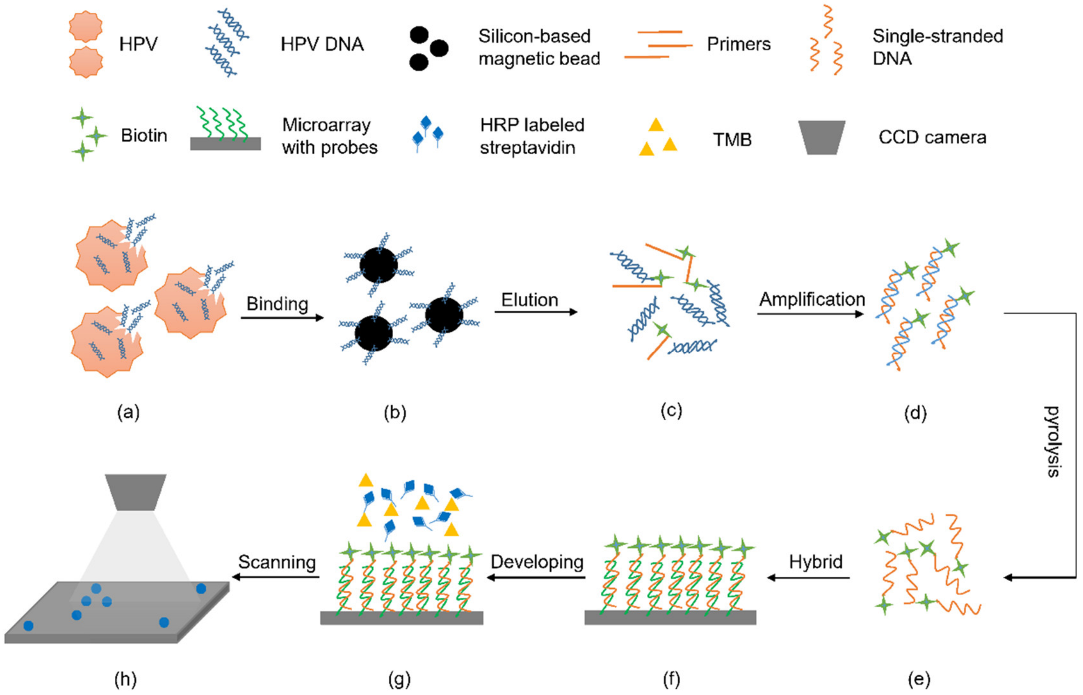

Figure S4. Schematic diagram of the principle of detecting HPV. (**a**) Lysis the virus to release viral DNA. (**b**) Silicon-based magnetic beads bind viral DNA. (**c**) After rinsing and elution, the nucleic acid bound on the magnetic beads is separated, and add reaction system containing primers labeled with biotin at the 3'end. (**d**) After PCR amplification, the products are also labeled with biotin. (**e**) The amplified products are pyrolyzed into single-stranded DNAs at high temperature. (**f**) The single-stranded DNAs are hybridized with the microarray with specific probes. (**g**) Add HRP-labeled streptavidin and reaction substrate TMB for color developing. (**h**) CCD camera scans and analyzes the test result.

Table S1. Primer sequences of 24 HPV genotypes and internal reference (GB).

Name	Primer Sequence (5′-3′)
L1 Forward primer Group 1	CGTAAACGTDTHCCMTATTTTTT
L1 Forward primer Group 2	CGTAAACGTATTCCCTTATTTTT
L1 Forward primer Group 3	CGTAAACGYTTWTCATATTTTTT
L1 Forward primer Group 4	CGTAAACGTRTKCACTATTCTTTT
L1 Forward primer 44	CGTAAACGTGTTTCCTTGTTTTT
L1 Forward primer 53	CGTAAACGTATTCCCTATTTTCTT
L1 Forward primer 68	CGTAAACACCTTCCTTATTTTTT
L1 Forward primer 73	CGTAAACGTCTGTCATATTCTTTT
GB Forward primer	GAATAACAGTGATAATTTCTGGG
L1 Reverse primer Group 1	ACTCTAAACACYCTATACTG
L1 Reverse primer Group 2	ACYCTAAAYACTCTGTATTG
L1 Reverse primer Group 3	ACCYTAAATACCCTGTATTG
L1 Reverse primer Group 4	ACCCTAAAHACYCTATATTG
L1 Reverse primer 42	ACTCTAAATACTCTGTACTG
L1 Reverse primer 58	ACCCTAAAGACCCTATACTG
L1 Reverse primer 81	ACACGAAACACCCGGTACTG
GB Reverse primer	GAAGATAAGAGGTATGAACATGA

Table S2. Probe sequences of 24 HPV genotypes.

HPV Genotype	Probe Sequence (5′-3′)
6	TTC CAT AAA ACG GGC TAA CAA A
11	ACT CTA TCA AAA AAG TTA ACA A
16	AAA CCT AAC AAT AAC AAA ATA TTA
18	GGT GGC AAT AAG CAG GAT A
31	AAA TCT GAC AAT CCT AAA AA
33	AAA AAT CCT ACT AAC GCT AAA AAA
35	AAA ACA AGA TTC TAA TAA AAT AGC A
39	TAA AGT GGG TAT GAA TGG TGG T
42	CAA AAA GGC CAA ATA AGA CA
43	CCT TAA AAA TTC CTC TGG TAA AA
44	ATA CGA CCA GCA AAC AAG AC
45	ACC TAA TGG TGC AGG TAA TA
51	TAA AAC CTC AAC GCG TGC T
52	AAA ACA CCA GTA GTG GTA ATG G
53	CAT TTC TAA ATC TGG TAA AGC A
56	AAG GAC AAT ACC AAA ACA AAC A
58	CCA TCA AAA GTC CCA ATA AC
59	GGT GGT AAT GGT AGA CAG GA
66	CAA ATC TGG TAC CAA AAC AAA
68	TTA AGG TTC CTA TGT CTG GG
73	ACG TTT TTG AGA ATC CTT GA
81	GGG TAC CAA TAG TTA ATG TAC AA
82	TAT TTC AGC ACG TGT ATT GG
83	TTT TTC CTT GAC CAT TAA C

Table S3. The remaining reagents contained in the matching detection kit.

Name	Component
Proteinase K	Proteinase K
Lysis buffer	Guanidine hydrochloride, MOPS, Tween
Magnetic beads	Silicon-based magnetic beads
IPA	IPA
Washing buffer I	Guanidine hydrochloride, MOPS
Washing buffer II	NaCl, MOPS, Ethanol
Elution buffer	Tris, EDTA
Membrane treating solution	NaOH
Water	Purified water
Hybridization washing buffer	SSPE, SDS
Color developing solution A	Urea peroxide
Color developing solution B	TMB
Positive control	HPV16 plasmid, GB plasmid, Tris, EDTA
Negative control	Tris, EDTA
